# Dr. Tavares, on Peruvian bark in Gout

**Published:** 1804-08-01

**Authors:** Joseph Adams

**Affiliations:** London


### To Dr. BRADLEY.

SIR,

IT must give every lover of his profession sincere pleasure to find, that those diseases once called the *opprobria medicorum* are now boldly and candidly investigated. If we are still undecided as to remedies, we may at least not be afraid to enquire after laws; and when these are established, we have rational grounds to proceed on.

Since the days of Sydenham, physicians seem to have been afraid of Gout. Some unfortunate events which followed what were considered as cures, very much increased the apprehension of interfering with that formidable enemy. The utmost that has been usually ventured on was to protect the stomach. Dr. Cadogan indeed exhibited a bolder kind of practice; but his treatment was for the most part so indiscriminate, his promises to his converts so unguarded, that his theory soon fell into discredit.

As soon after my arrival at Madeira as I could avail myself of that leisure which a new situation afforded me, a fit of the gout induced me to examine my papers on that subject, and to recollect all I had observed of the disease. And here I can bear testimony to Mr. Wadd's claim to the use of cold water at least ten years ago, and to that of his late father in law, Dr. Waymann, near twenty years before him. When all my materials were prepared and almost arranged for the press, I found the disadvantage of living so far from the intercourse of men engaged in experimental enquiries. The analyses made of gouty calculi was in direct opposition to one part of my theory, and of course determined me to lay aside my work. It however gives me much satisfaction to find, that the practice in that disease is becoming every day more bold; and that even so illustrious a character as Heberden has sanctioned bleeding in acute gout. Those who peruse Sydenham with care, will find he is by no means chargeable with the practice which so generally obtained after his time. On the contrary, there are cases in which he advises bleeding; and it was his constant custom occasionally to purge himself.

These remarks are intended only to introduce a work, which, as far as I know, has not hitherto appeared in England. It is the production of one who holds the important office of *Archiatros of Portugal*, and containing some valuable facts which he witnessed on himself as well as others, is is particularly entitled to notice. The work is written in Portugueze and Latin. The former, a language little cultivated by the profession; and as the liberality of the times, and the miscellaneous contents of your Journal, encourage the diffusion of knowledge, the following translation from the two languages may not be inconsistent with your plan.

"*Observations on the safe and efficacious Use of Peruvian Bark for the Cure of Gout, by* FRANCISCO TAVARES, *First Physician of the Kingdom of Portugal, &c. &c.*

That celebrated investigator of the nature of diseases, Sydenham, long since suspected that the Peruvian bark, whose virtues exceeded most other then known articles in the Materia Medica, might hereafter be found useful in gout, which had hitherto withstood the force of so many remedies. Boerhaave, and his illustrious commentator, seemed to assent to this opinion; but without adding any thing of their own, contented themselves with a respectful reference to the English Hippocrates. Though the celebrated Held, in the Ephemerides of Natural Curiosities, left several important facts concerning the safe use of this remedy in gout, yet unfortunately they stand unsupported by any other authority; on this account, Murray not having prosecuted the enquiry experimentally, dismisses the article with a few words on the virtues of bark in the gout.* Perhaps cautious physicians, dreading the fatal effects of the imprudent use of bitter remedies, which have been handed down to us from the ancients, were fearful of trying bark, and willing to leave the subject untouched.

It has been the fate of the most valuable remedies to be most opposed. Sometimes the ignorance of physicians, and their want of diligence in attending to the exhibition of these medicines, or their impatience in being disappointed in their first and perhaps ill-directed experiments, have been the cause of this opposition. I am prepared to expect the same opposition to the use of bark in the gout, though for more than eight years I have experienced the advantage of it in my own person, and have confirmed this experience by general practice.

On this occasion I feel it a duty to express publicly my acknowledgments to Sen. Bento Joachim de Lemos, once my pupil, but now Professor of Medicine in the University of Coimbro. Urged by our ancient friendship, he communicated

* Appar. Medic. tom. i. p. 603.

municated to me his observations, the source of which will soon be related, and entreated me by repeated letters to make use of bark, to lessen the violent and frequent attacks of a disease which had tormented me for sixteen years.

I confess that for some time I neglected the advice and wishes of my friend, nor shall I conceal that I was fearful of the practice, recollecting the cautions of Galen, Celsus, Aurelianus, Gaubius, Van Swieten, Cullen, and others, against bitter remedies in the gout. Thus divided on one side by terror, on the other by the well known abilities of the Professor, and my confidence in his probity and friendship, I willingly reflected that the bark was not only free from the bitterness of the once celebrated Portland powder, but that it was also serviceable in some diseases which have a strong analogy with gout, and that it even possessed properties which promised relief to that complaint. From revolving all these circumstances, I flattered myself, that without a perfect cure, I might at least lessen the violence and frequency of the paroxysms, advantages which my fellow-sufferers only can justly appreciate.

Under this persuasion, which has been confirmed by experience, I have spared no pains to establish the usefulness of the remedy by every authority I could collect; I shall therefore first relate the observations of my friend, explain with candour what I have experienced in my own case, and, lastly, since, to use the words of Celsus, the facts have not been discovered after, but have led to the theory, I have thought it right to add the reasons for this agreement, the necessity of the practice, and an account of such remedies as have probably assisted the use of the bark.

Case I. giving an account of the circumstances which led to the discovery.

Professor de Lemos being called to visit a Cistercian monk in the gout, was requested either to cure him or to cut off his legs. To this my friend briefly replied, that it was as difficult to do one as it was easy to do the other, and that he could prescribe nothing but time, patience, and diet, with a very few remedies to be cautiously exhibited during the paroxysm. The village surgeon, scarcely deserving the name of a barber, was present, and had promised to cure the patient. He smiled at the prudence of the Professor, and again renewed his promise. Dr. Lemos took leave of his patient, and was soon afterwards consulted sulted concerning a purge, which the barber had prescribed for their patient. This consisted of half a drachm of rezin of jalap, the same quantity of scammony mixed with half an ounce of diacodium, for a single dose. The Professor with difficulty suppressing laughter, astonishment, rage, and impatience, at length sufficiently composed himself to reply, that a fourth part of such a dose would be a dangerous remedy. What quantity the monk took, never was ascertained; but after the purge a drachm of powdered bark was exhibited every hour, so that in the night and day, two ounces were taken. Dr. de Lemos renewed his visit, expecting to find the monk in a worse state than before; but what was his astonishment to find him walking on crutches, and two days after quitting his house. This fact suggested to him the exhibition of a purge consisting of ℥j or ℥jß of Epsom salt, giving in the mean time the bark in the above quantity and doses. Of the monk he never after could gain any intelligence.

Case II. The Rev. F. I. de T. director of the apothecary's shop at the royal hospital of Coimbro, about forty-six years old, fat and sedentary, having in the year 1796 suffered for ten days the first gouty paroxysm in his great toe, felt a repetition of the disease about three months afterwards. Prof. Lemos was consulted on the third day, and prescribed the purging salt and Peruvian bark in the manner above related. The pain was so much lessened, that on the following day the patient left his bed, and on the immediately succeeding day walked without difficulty. About a year after he had a fresh attack, and derived the same benefit from the same remedies. He continued free from the complaint for two years. As soon as the first symptoms of the paroxysm appeared, he took his purge, and afterwards only 3 drachms of powdered bark. From these, if he did not derive the desired effect, he at least found that his pains were not increased, and on the following day they yielded to 6 more drachms of bark. Hence it is evident, that to produce the desired effect, a large quantity of bark is necessary. From that time (1799) to 1802, he has felt no return, except that recovering from a fever he found a pain and swelling in his knee, which the bark relieved.

Case III. I. A. fat and sedentary from his occupation, from 45 to 50 years of age, was for many years gouty, the paroxysms continuing never less than twelve or fourteen days, and sometimes longer. Being treated according ing to the above plan, in the three last paroxysms, though his stomach would not bear such repeated doses of bark, yet he always obtained relief from his pains, nor did any one of these fits detain him longer than four days in the house. Though he was accustomed to suffer two attacks in the year, yet since the use of the bark he has been visited in the space of eight years with only the three above mentioned. This winter (1802) he has suffered much by the catarrhal disposition of the season, but has been free from gout.

Professor Lemos has many other cases, of which he kept no memoranda. I shall subjoin those under my own observation.

Case IV. I. da C. a shoe-maker, having gone through his first gouty paroxysm in his great toe, three weeks afterwards was attacked in the hand. He took the bitter salt and bark in the manner described, and the pain ceased within twenty-four hours. By my advice he discontinued the use of the bark. One month afterwards, feeling a troublesome sensation in his foot, he returned to the remedy, and after the third dose the uneasy sensation ceased, the motion was free, and his health restored.

Case V. The author's own. At the age of 33 years, being advanced to the rank of public Professor in ordinary in the University of Coimbro, the close application and confinement such an office required, soon brought into action that hereditary disposition to gout which I had derived from my father, and which I had probably hitherto only prevented by muscular exercise. After a severe rheumatism, I suffered for the first time (1786) acute gout in my instep; this continued about fifteen days, after which I remained well for a twelvemonth. In the following spring, however, the paroxysm returned, and two years afterwards, with the same regularity. After this last paroxysm I was teased with want of appetite, and sometimes vomitings, which I attributed to indigestion, being accustomed to lecture every evening soon after dinner. For this complaint I repaired to the Caldas, drank the waters, and remained two years free of gout. At the close of 1790 the gout returned, and again in the following spring. In the succeeding year, 1792, the fit occurred in an advanced state of the summer; it was preceded by a bilious fever, and continued a month. Though at this time, by orders from the Superior of the University, I had changed my plan of life from sedentary science to active employment,

 yet yet at the end of two years the gout returned with as regular a paroxysm as before.

Hitherto, my feet only had suffered; but in the year 1797, for the first time, the disease passed from my feet to my knee, and from that time I do not recollect a single paroxysm, which after three or four days did not take the same course. Nor were the attacks as before, confined to once in a year: but by the recurrence of a second attack the parts were during the intermediate space painful, and unfit for their necessary functions. I now became dispirited; and although my diet was strictly regular, yet frequent indigestions occurred whenever I omitted muscular exercise, either from the duties of my office or merely from melancholy. I had suffered the longest paroxysm of all in the year 1800; and at length, on the 11th of September, 1801, having slightly strained my left foot, it became the seat of gout; afterwards my right foot, and alternately my knees and feet, for three months. On the decline of the paroxysm, weary of so much suffering, I took a solution of rezin of guaiacum in gum arabic, syrup, and cinnamon water, from which I derived relief sooner than in my former paroxysms. The effects of this remedy were a gentle perspiration during the night, and one or two bilious stools in the day: and though I did not acquire the perfect use of my limbs, I could walk with a crutch.

On the 28th of October, from a very slight cause, my feet were attacked, and my knees were more painful than at any former period. On this occasion I had reason to believe that the solution of guaiacum shortened the period, for in the space of four weeks I found myself restored. At the end of that time, without any previous notice, the joint of my right toe became painful but very slightly, my knee began to be affected in the same way, and my leg to swell. The weather was intensely cold and damp; the wind blowing furiously, and varying from north to south. There was also an epidemic catarrh, the effects of which I felt with pain in the head. At length, on December 27, at two o'clock in the morning, commenced an acute pain in my foot, and my knee was relieved.

It was now that the recollection of my recent severe sufferings reminded me of the advice of my friend. I began the bark, and in thirty-six hours took 18 drachms of the powder; no inconvenience in my stomach followed; no increase of pain, no incapacity to move, though my foot was firm on the ground; no sensation of weight in my leg, nor any of that inquietude or impatience peculiar to gouty subjects.

On

On the 29th of December, by the assistance of a crutch I could fulfil my necessary duties, moving indeed slowly but without perceiving any weight in my foot, or swelling of my leg. I laid aside the bark for two days, and after this I took fourteen drachms, and in eight days swallowed four ounces, remaining only two days at home. Aware that the bark in large doses usually purged me, I omitted the previous dose of Epsom salt. In this I was not mistaken, for after the third dose of bark, followed three copious bilious evacuations. For this reason, within the twenty-four hours I took ten drachms. I now laid aside the bark for four days more, and afterwards took two drachms each day, or sometimes, instead of the powder, took a mixture of bark and wine, varying the form, that the remedy might not lose its effect. For some time a slight pain continued, for which the cold and wet weather were sufficient to account; however, I never failed to go abroad morning and evening; and excepting the uneasiness that attended first leaving my bed, could accomplish the whole with little or no difficulty. But this pain, however slight, continued even to the month of February (1802) which induced me to repent of my obstinacy in not purging myself freely at the beginning of the paroxysm; I therefore took 20 grains of jalap and 8 of calomel. After this the pain almost entirely ceased, nothing remaining but some degree of debility, which I attributed to the frequent repetition of paroxysms and the want of exercise in the open air, which the inclemency of the season prevented.

From the 22d and 24th of February, being clear days, with a heat equal to that of Summer, I took the exercise of riding. The following days were cold and cloudy with a north-east wind, which I have reason to remember, for without any other cause to which I could ascribe it, I was attacked by a fresh pain in my great toe at two o'clock in the morning. I have no doubt that it would have increased, if I had not instantly had recourse to what I now considered a sheet anchor. Scarcely had I taken the third drachm of bark when the pain became milder, though I had prepared myself to expect a violent paroxysm, having already found the veins of my foot and leg tumid, an erysipelatous efflorescence, swelling, spasmodic contractions of the muscles of the leg, and cramp in the foot, accompanied with the usual impatience and inquietude, fever, shiverings, and all these exasperated by a fifth attack of the catarrh before mentioned. The pain was so much relieved that I could rest on my foot and walk out, nor did any other of the symptoms remain. On the following days, though very cold, I continued to go out, halt indeed in my first steps; but after a few minutes the very act of walking almost fitted me for it. On the first day I took a drachm of bark every hour till I had consumed an ounce; after this, passed the night well, sleeping soundly, my foot being so easy that no inconvenience was felt excepting from some aukwardness in its position. On the following days I took another ounce of bark, and afterwards desisted. I recovered my health in the beginning of March, excepting only a trifling swelling; and so sensibly am I impressed with the value of this remedy that, or the future, I shall never fail to have recourse to it, or to recommend it to others.

Case VI. Whilst preparing these sheets, I communicated the importance of bark in gouty cases to my worthy friend, Senr. Norberto Antonio Chalbert, Surgeon to the Royal Camera, a man of well known reputation, and of no common erudition or prudence. A few days after he was sent for by an old gouty subject in an acute paroxysm. This patient had frequently been under Senr. Chalbert's care, and no former remedies he could suggest ever lessened the fit, so as to reduce the period within the space of a month. He prescribed however a purge on this occasion, and in the afternoon began with the bark in the manner above described, continuing it for the two following days till two ounces were consumed. On the third day the patient thought himself well enough to leave his house, without any considerable uneasiness.

Case VII. F. Z. formerly a dancing master, fifty years of age, has been afflicted with gout from the age of twenty-five. For this reason his mode of life is sedentary; he is lean and full of vivacity, yet has usually two paroxysms in a year, which continue for three months or longer. In the beginning of January 1802, he had a paroxysm which lasted till March, and on the 18th of that month the disease attacked his feet with more violence than before, passed to the elbow, and thence to the right hand; again to the feet, the ancle, the heel, the *tendo* Achillis, and the thumb. On the 6th of April I visited him, and found the foot swoln near the joint with an efflorescence, and painful when pressed upon: the right hand had not yet recovered its size or free motion. I ordered an ounce of Epsom salt, from which he had an easy and copious evacuation. On the same evening I advised him to take half an ounce ounce of bark before midnight, as he assured me his stomach was equal to it: however, after the third drachm, he felt some repugnance. But with the quantity taken he slept soundly, contrary to his usual custom, and was free from pain. In the morning the motion of his feet was more free, and his hand in such a state as enabled him to write me a letter, describing his situation. After taking three drachms more on the same morning, his stomach became uneasy, and he vomited a small portion of the last dose. I proposed longer intervals between the doses. He took a drachm four times a-day, and though I could not promise him such speedy relief, I trusted that the efficacy of the remedy would not be interrupted by the too great sensibility of his stomach. Though he had not taken quite two ounces of bark, yet on the following day he was so far relieved from pain, swelling, redness, and difficult motion, that he could walk like a person in health, and take his exercise abroad. For the perfect establishment of his health I advised a cold infusion of bark for a few days; this his stomach bore without inconvenience.

Case VIII. Having bruised my knee near the patella I began to feel threatening of gout, the pain gradually increasing over the whole articulation. Having applied three leeches to the bruised part, I took half an ounce of bark, and in twenty-four hours found myself perfectly well.

I omit several other cases; those already related are sufficient to encourage any one who wishes to make trial of this remedy.

By what has been said, it is evident that the advantages of the proposed method consists in cleansing the primæ viæ by a powerful cathartic, as soon as the pain commences, and afterwards in exhibiting the bark in the dose of a drachm from hour to hour. For the two first days an ounce should be taken daily; on the four following, half an ounce each, increasing or lessening these quantities according to the judgment of a prudent physician.—Whether the bark is given in powder (which is always desirable) or in infusion or decoction, the quantity given must depend on the patient's stomach and other circumstances.

### *The Author's Reflections.*

Whatever is worth the attention of the physician in his observations on gout, as to its nature, the progress of its paroxysms, or variety of symptoms, and also whatever has been observed of the bark, all contribute to recommend the latter above all other remedies hitherto proposed in the cure of this disease. Let us view these propositions in order.

As to the nature of gout, all authors agree that debility of the viscera, hereditary or acquired, producing indigestions, incomplete assimilations, and various complaints arising from the same causes, and showing themselves under a variety of forms, at length terminate in gout. It is evident, that all those causes (of which no physician is ignorant, and which it is unnecessary here to enlarge upon) which may produce or excite this debility in a subject predisposed thereto; in short, whatever may injure the nervous energy, and induce disease arising therefrom, may likewise excite gout. Hence, according to the variety of subjects and external circumstances, the disease appears under its regular paroxysms, anomalous, atonic, misplaced, retrograde, earlier or later, at greater or less intervals, changing its form, whilst its nature continues the same.

The ancient physicians always kept in view, that in curing the gout, the strength of the viscera should be restored: all recommended during the interval of the paroxysms, tonic, bitter, and gently stimulating remedies, even so far as to propose proper condiments to be taken with the food, in order to assist digestion. Whatever may be handed down to us from Celsus, Aurelianus, Ætius, Aretæus, Galen, and others, as servicable in this complaint, are bitter remedies, administered with due caution, with the view to strengthening the stomach and intestines, and by that means procuring a more perfect assimilation of the food, and a regular secretion, excretion, and distribution of the juices, by which the strength of the solids being increased with every assistance from diet, a firm state of health might be established. Nor has the opinions of the moderns greatly differed; all have proposed medicines with a view to the same indications. All indeed have selected such remedies as they conceived best calculated for such purposes; but none of the modern writers that I am acquainted with, since the discovery of the Peruvian bark, speak of its virtues in the cure of gout, given in the manner here proposed. Sydenham, who was the first that advised the use of it, gave it in the quantity of a few grains daily. To pass over others, I recollect that Cullen, after condemning the use of other bitters in this disease, recommended the Peruvian bark with the same intention as Sydenham, merely as stomachic and strengthening, in the same same way as he proposed steel. Gotf. Held was the only person who, as early as 1714, considered bark as a specific against the gout. Not satisfied with the small doses proposed by Sydenham, he used it in somewhat larger quantities. After objecting to Sydenham's small doses, he adds, "Ego vero hactenus in praxi mea hunc corticem non granis sed plerumque ad scrupulum unum, vel drachmam dimidiam ordinavi, et illum quidem preservativè semel, vel bis in hebdomada; curative vero, malo jam appropinquante, mane et circa noctem assumere jussi; et sine topicis, tumor et dolores brevi remiserunt; febris in paroxysmo cum calore presens mitigata, et appetitus ciborum alias imminutus, vel plane abolitus resuscitatus, vel integre servatus, imo paroxysmus omnis brevi sublatus. Cortex enim noster ob blandum saporem amarum, fermentum gastricum vitiosum idque vel acre biliosum, aut v scidius acidum immutat; simulque, quod magnum in podagra beneficium, alvum apertam servat; sanguini quoque vigorem conciliat, ejusque acrimoniam corrigit; ac fibras tendinosas nerveas roborat, et solatur, sicque spasmodicas surarum, et aliarum partium contractiones et antevertit et abigit: anodyna etiam virtute pollens et blandum somnum, inducit et dolores demulcet, nec minus cardiacum est, et collapsas vires restaurat. Uno verbo, Cortex Peruvianus in podagra divinum est remedium, quod multijuga experientia edoctus certò et firmissimè asseverare non nequeo." (Ephem. N. C. Centur. 3 & 4, Obs. 170, pag. 384.) It is much to be regretted, that this high road, so accessible to the most common observer, should, with many other equally valuable things, have become almost obliterated from the memory of man. The foundations of the healing art must be constructed not of visionary systems and theories, but of observations properly instituted, carefully repeated, and firmly established.

But though we should not be able to ascertain by authority the safety of giving bark in the gout, I trust those analogies which I shall now explain, will be sufficient to satisfy the attentive observer. If we once establish the use of bark in other similar diseases, we shall cease to wonder at its usefulness in this. Every attentive practitioner, and still more, every unhappy sufferer, will bear witness that the gout preserves regular periods in its paroxysms, not only respecting the season but the hour of invasion and remission; hence appears a ready analogy between this disease and the intermittent fever. Every one knows that the close of January, or the beginning of February, as well as the advanced state of autumn, are seasons most dreaded by the gouty, as well as those in which we are to expect intermittents. It is also well known, that the autumnal paroxysms of each disease are the most obstinate, and that without the greatest caution, there is also most danger of a misplaced gout.

The gout, in its first attack, arrives at its most painful stage between two and three o'clock in the morning; on the following and succeeding days, the paroxysm returns about the same hour, going off with perspiration of the inflamed joint, or of the whole body. Oftentimes it imitates the exacerbations of the single or double tertians; and after the morning paroxysm another will regularly follow about four in the afternoon. In almost all my gouty attacks I have suffered this second or evening paroxysm with so much regularity, that I have constantly expected an increase of pain at seven in the evening, which should last till ten. Since the use of the bark this second paroxysm has never occurred.

It can hardly be disputed that the gout terminates after a certain period and number of paroxysms, varying according to circumstances; and also that, like the intermittent fever, every one of these fits finishes with a perspiration, having a sour smell, with turbid or bilious urine with a white lateritious sediment. It is also right to remark, as it may serve to strengthen the analogy, that gouty people are rarely if ever afflicted with ague; at least, among the numberless subjects of my acquaintance, I do not recollect one who caught the ague even when epidemic. Still further, the inhabitants of low and damp situations are more subject to gout than those who, in more elevated spots, enjoy a dry, clear, and better ventilated atmosphere. In the same manner as intermittents, gout follows the changes of season and vicissitudes of temperature; nay, it often precedes and foretells them, so that when cold, dampness, or a cloudiness are present, or approaching, not only is the present paroxysm exasperated and prolonged, but gouty subjects at that time free from the complaint, will be sensible of wandering arthritic pains, and other inconveniences; not uncommonly, these will prove the immediate cause of a fresh attack. Stoll, among the anomalies of the gouty fever, mentions vagam intermittentem, vagam remittentem, veram obscuram tamen ac veluti inchoatam intermittentem, quæ instituto paroxysmo cessat; and lastly, (after establishing the analogy in their attack and termination, between the bilious fever and gout) he considers it among the the properties of gouty matter, that frequently an intermittent coming on a well treated gout, will prove its cure; in the same manner that an ill treated bilious or remittent fever will terminate in gout. It should also be remarked, that the celebrated Storck, in his Diss. de Pleuritide, suspecting this analogy between a truly inflammatory disease and the intermittent fever, proposed not long since treating pleurisy with extract of bark. In the acute rheumatism, a disease very similar to gout, Dr. Saunders exhibited with advantage, after the seventh day from the beginning of the disease, bark in conjunction with anti-phlogistic remedies, (Observations on Red Bark) probably induced by a similitude between that disease and the double tertian; nor does Cullen object to this practice, (Elements of the Practice of Physic, §. 469.) Considering then that the commencement and progress of gout is very similar to those of the intermittent fever, we may fairly infer that the bark will be useful in lessening the violence of the paroxysm in one as we find it actually does in the other.

The best authors advise in the worst species of intermittents, even attended with inflammatory symptoms, to give the bark rather too early than omit it too long; and if ever it disappoints them, they impute it to a too sparing exhibition of, or to the bad properties in, the remedy, finding by experience that no danger attends the giving a larger quantity. Nor is it possible to cure the worst species of intermittents, unless a large quantity is given as well in the interval of remission or intermission as during the paroxysm itself. To give the authority of innumerable writers on a fact so well established, could answer no other purpose than to show the writer's erudition. Why then should we not exhibit bark in the interval as well as during the paroxysm of gout, if we can diminish the severity of its pains? It may be dreaded lest, by suppressing the attack, the disease should become wandering or retrograde; but observation proves, that notwithstanding the pains and some other symptoms are evidently lessened, the paroxysms indeed are shortened but not extinguished. It is worth remark too, that in those cases where the gouty action is imperfect in the joints, it is thought necessary to preserve the stomach from the deposition of what is called gouty matter, by the use of tonics, such as bitters and stimulants; and among the rest, bark infused in wine is particularly recommended. It is therefore reasonable to hope, that at the access of a gouty fit, whether regular or atonic, bark is so far from being likely to obstruct the endeavours endeavours of the system, that, given in large doses, no remedy seems better calculated to assist them.

Another still closer analogy has gout with diseases of the nerves, in which bark is a wonderful remedy. Not only the nature of the symptoms but the causes are common to both. Prostration of appetite, nausea, dyspepsia, vomitings, flatulence, pains, spasms about the region of the stomach and in other parts of the body, the belly at one time lax at another constipated. Hypochondriac symptoms, such as fearfulness, uneasiness, impatience, besides many other complaints, are only those nervous symptoms which often precede a gouty paroxysm, or harrass the patient as the fit declines. The facility with which the gouty pain shifts from one part to another, leaving the first free, shews a nervous affection afterwards connected with local inflammation. All modern authors admit the importance of bark in nervous complaints. These are the principal diseases described by Tissot in his remarks on the diseases of studious men, and of such as are addicted to excessive venery; and for these, in common with other authors, he recommends the bark. The very causes above mentioned, viz. close application, a sedentary life, and excessive indulgences, by the weakness they induce, become the exciting causes of gout, and should seem to require similar remedies.

Peruvian bark holds the first rank among bitter remedies. 1st. Because, to use the language of Murray, it strengthens most without producing heat. 2dly. Because it may be accommodated to every temperament, as daily experience shows in the cure of intermittents. 3dly. Because, by long continued use, it produces no atony in the stomach, which is not the case with other bitter remedies. Physicians have long since given up the vulgar dread that bark, in too large quantities, produces obstructions in the viscera, scurvy, and other complaints, which the observation of later ages prove to be unfounded, and that the general error has been not in the excessive but the sparing doses of that remedy. We ought not to wonder if, for the cure of so stubborn a disease as gout, a large quantity of bark should be necessary, as we all know that obstinate agues require as much or still more. Nor ought we to be surprized if the gout returns at determined distances of time, since the same happens not only with quartans, but with the milder tertians; and certain it is, that there are persons in whom ague, however often cured, seems to return occasionally like a constitutional disease. Still further, it has has been shown that bark, given in smaller doses than will consume two ounces in the first two days, will be less successful, though not without its effects. From all which, it follows, that whatever propriety there might be in the cautions against the improper exhibition of bitter remedies, there can be no danger of the use of bark, even in the large doses above prescribed.

On the respectable authority of Sydenham, we might apprehend the exhibition of a purge in the beginning of an attack. That celebrated author entertained this apprehension even at the close of the paroxysm, and was fearful, during the intervals, lest purges might hasten a return. He appears however, afterwards, to have become less fearful, *(de mictur. cruenta)* and advises that, after a purge, an opiate should be given, to lessen the nervous irritation excited by the former. I might further assert, that many gouty paroxysms have, in myself, yielded to spontaneous bilious evacuations; and without recommending such a practice, am persuaded, that by promoting such evacuations, some paroxysms have been shortened, and others prevented. Laying aside the almost obsolete notion of gouty matter, which by such stimuli may be transferred to the intestines, it must on all hands be allowed, that by such evacuations the disposition to plethora must be lessened, any accumulation in the bowels removed, and the bark produce its full efficacy. The experience in my own case seems to confirm this. Though I found some relief from the bark only, it was less than others had done, nor was I restored to health till after a brisk purge.

Boerhaave* was not afraid of purges in gout, and particularly recommends those which he calls serous, and which he advises to be quickened by mercurial preparations. He proposed to his old arthritic friend, Bassand, the powdered scammony with unwashed calx of antimony. Van Swieten was so well satisfied with this doctrine, that in his commentary on the passage he does not scruple to recommend it, after examining the foundation of the practice, and adding the result of his own observations. There are not wanting gouty subjects, who conceive they have escaped the return of paroxysms by taking a purge every week. Dr. Joseph Quarin (Animadvers. cap. 15, p. 290) observes, that the body should be occasionally opened during

* Aphorism 1270.

during the paroxysm with the saline mixture of rob. sambuci, whenever the previous mode of living has been too free, if the tongue is foul, and the heat considerable.—Andrew Szootes* recommends eccoprotics, such as lenitive electuary with sulphur, some grains of rhubarb with manna and tamarinds, when the previous symptoms of gout appear, in its first, and in all its subsequent stages. Hoffman gives it as an axiom,† in all cases of pain, to purge before the exhibition of any other remedy. By these means, wherever the pain is situated, it is rendered milder, as, he asserts, he has repeatedly experienced in his own person.

My friend, Professor Lemos, very prudently exchanged the violent drastic proposed by the barber for Epsom salts, which he gave in a sufficient dose gently and copiously to cleanse the primæ viæ, and the better to ensure the efficacy of the bark; yet there are people with whom these salts disagree, producing nausea and vomiting instead of the desired effect. A mixture of tartarized infusion of sena with the salts, or with a small dose of tincture of jallap, will answer every purpose; and should the system seem too much agitated by this medicine, it has been before remarked, that the antispasmodic powers of the bark will be likely to relieve not only this inconvenience, but even that restlessness, impatience, and irritability of temper so characteristic of this disease. In my own case I have remarked, and it often happens in others, that the first doses of bark prove purgative; but notwithstanding this, I found the advantage of cathartic remedies; and the best practitioners are of opinion, that in intermittents the bark is more efficacious if preceded by laxative remedies.

There are not wanting such as maintain, that the inflammatory diathesis is a contra indication to the use of bark. But besides the authorities already produced from the best writers, and that the celebrated Storck treated pleurisy with extract of bark, we may further remark, 1st. that the pain from gout precedes the inflammatory symptoms nearly twenty-four hours; 2dly, that even the gouty inflammation is no objection to the use of bark, because this inflammation terminates only by resolution or desquamation, never producing suppuration, scirrhus, nor gangrene;

---

* Collectio Dissertat. circa morbos chron. Stollii, t. 1.

† Medendi Rat. Systema, tom. iv. p. 2. S. 2, c. n.

grene; in these respects approaching nearer to the erysipelatous. In these cases, Dr. George Fordyce (following Haller,* who found benefit from bark in his own person, when at the same time afflicted with erysipelas in his head and gout). asserts, that bark may be given with advantage, urging, that in the erysipelatous malignant sore throat, bark should be given in large doses. He afterwards extended the use of this remedy to all erysipelatous inflammations immixed with phlegmon, and even found it advisable to give it in the dose of a drachm every hour, if the stomach would bear it.†

Some advantage may arise from hinting at such other remedies as may increase the efficacy of bark, and hereafter bring it into more general use. No preparation is better than the powder during the paroxysm; but to patients of an extremely irritable stomach, the cold infusion may be equally servicable. Cullen and Lewis both inform us, that this may be made in less than twenty-four hours. If the powder is taken, it should be diluted in water, or being only wetted, it should be placed on the tongue, and swallowed by the assistance of water. Other changes may be made, according to the discretion of the physician.

When the inflammation is very violent, the application of leeches to the part may very much assist these other means. Before I was acquainted with the virtues of bark, I have frequently found the advantage of leeches in relieving the pain, when its violence induced me to expect a proportionate inflammation. But only in a single instance did I find the paroxysm shortened, though the bark has constantly produced that effect. There is reason to hope that both these assistances, preceded by a purge, may produce the best effect.

It has been already remarked, that the solution of guaiacum has shortened the paroxysm. There can be no objection therefore to the use of it at night, to produce a gentle perspiration after taking the bark. In order to fulfil every indication, it may be right to consider what diet will be the most suitable. This may done in a few words. Light nourishing food, easiest of digestion, gently spiced, and taken according to the powers of the stomach, and previous exercise: flesh not too strong at dinner; a sparing

---

* Disput. ad Morbor. Histor. & Curationem. No. CLII. tom. vi. p. 836, towards the bottom.

† Transactions of a Society, &c. page 290 to 293, Art. xvi.

sparing and vegetable supper not protracted too late at night. It may be a question whether fish is proper for gouty subjects. If not oily or hardened by long salting, or dressed so high as to excite an unnatural appetite, there can be no objection to their use. On all these subjects, the common cautions have been so often repeated as to render it unnecessary to say more. Twelve years ago, I found fish so very hurtful to my own constitution, that before the conclusion of the first week in Lent, I was seized with gout, and found it necessary to return altogether to flesh. On the day, or the day succeeding a dinner of fish, I found threats of a gouty attack; but since the use of the bark, I have not only derived the advantages above mentioned, but during the Lent of this year, 1802, have confined myself to fish without any inconvenience. Those who are accustomed to drink wine with moderation, need not alter their habit, and for others a small quantity of Port may be serviceable. Water more or less cold, according to the season, but still cold, is very serviceable, taken in the morning fasting. Thin chocolate is the best breakfast, and a small quantity of coffee should be taken after dinner. Tea, and other liquors of that kind, may be thought hurtful by weakening the stomach; on this subject, custom and experience must be our guide. Sleep should be moderate, and at proper seasons: the patient should retire early to a bed not too soft, which he should quit early in the morning. He should avoid those evacuations which are not indicated. He should cultivate a cheerful mind, avoiding as much as possible those reflections which may sour his disposition, and also too close application. In short, the common rules for preserving health should be attended to in a particular manner by gouty subjects.

Excepting the customary injunction for what are called the non-naturals, the patient should avoid all medicines for a month or two, that he may be the more susceptible of their effects. At the end of that time it may be advisable to take a gentle purge, and afterwards an ounce of powdered bark, or a pint of the vinous infusion, or somewhat more of the cold watery infusion, or a saturated decoction for four days. Those who are troubled with phlegm, may add a small portion of Aristolochia, or wherever a stimulant is necessary, the powdered Aron may be a useful addition; or, if the stomach seems averse to these remedies, the steel wine may be exhibited alone or with some spirituous compound. The vitriolic acid with alcohol (liquor anod. mineral. Hofmanni) may be given in sugar sugar with advantage. After this, the patient should abstain from remedies, and attend only to diet.

Frequent exercise is the most important consideration during the interval of the paroxysms. It may be varied according to circumstance, by walking, riding, or gestation. It is certain, that on the decline of the paroxyms, they are much shortened by exercise. Sydenham advises it; reason and experience confirm his advice. I can with truth attribute the shortening of several paroxysms to using my feet whilst that use was still painful. They always mended as I proceeded, and long rest constantly induced a return of pain.

Having thus faithfully translated all the facts offered by Professor Tavares, your readers will be satisfied with a more general account of his remaining reflexions; or such as wish more minutely to peruse them, will refer to the original.

Cold bathing, as assisting the purposes of the bark, is advised either in the sea or at home. If the latter, the author prefers ablutions with sponge, or if bathing is preferred he advises only a single immersion; if in the sea, the time of bathing should be left to the sensations of the patient. The usual cautions are added.

Under all these means, Dr. Tavares only promises an alleviation of a disease for which the patient must have a predisposition. By this we conceive is meant, that the predisposition being a part of the original constitution, cannot be altered by remedies. But by his own and some other cases, it appears that not only the paroxysms may be shortened, but their return much protracted.

Some remarks follow on the importance of accurately distinguishing the disease, the state of the patient under it, and the necessity, in some instances, of producing a full fit for the relief of the whole frame. In all these cases, he wishes the patient to take the advice of an experienced physician, and, as an illustration, adds (in a note as it probably occurred after the work commenced) his own case in the year 1802.

On the 9th of June, without any previous apparent cause excepting the great and sudden changes of weather, he suffered a violent cramp in the whole course of *musculus peronæus longus* for near twelve hours. As it went off, a shining redness appeared for three days. As this vanished he felt restlenesss, anxiety, palpitations of the heart, impatience, slight vertigo, and lastly a sense of pain and heat coming on in both knees; all the former symptoms (the foreforerunners of a gouty paroxysm) were diminished. Suspecting the beginning and progress of a paroxysm, he waited patiently for its more complete formation, continuing his accustomed occupations, and using no remedy. On the 16th day of the month, the uneasiness in the knees going off, an obscure pain in the wrist threatened gout in that part; but suspecting a sluggishness in the attack, he took only two drachms of bark, either to throw out the disease or stop it, avoiding a repetition of the remedy lest he should interrupt the efforts of nature. Two hours after midnight of the 17th, a manifest and violent gout appeared in the wrist. After it had raged for sixteen hours, and whilst the inflammation was still high, and a white lateritious sediment appeared in the urine, he began taking the decoction of bark in the manner advised for intermittent fevers. After the third dose, a remission of pain began, which gradually lessened, so that he enjoyed a good night's rest. Neither the swelling nor inflammation subsided, but the motions of the hand and arm were now only impeded by their increased volume and not by pain.

After this follow some remarks on the different kinds of bark, a subject which has been amply discussed at home. The author also suspects that the white willow bark may be found efficacious in gout, as he experienced its usefulness even in obstinate intermittents.

Particular respect is due to the observations of an arthritic physician, on whatever relates to his own complaint. The use of opium, in all its preparations, he condemns from his own experience and his conversations with his brethren. The necessity of gradually augmenting the dose is strongly urged, and inconveniences thence following to the nervous system, by which the irritability of the constitution is much increased. Warm bathings of every kind are objected to for similar reasons, excepting the warm sulphur and sea or salt-water tepid baths. Even these the Professor objects to during the paroxysm. As to pediluvia in warm water, mixed with marine acid, though recommended by Dr. Rowley during the gouty fit, our author thinks should rather be used during the atonic or indolent stage.

The whole concludes with expressing, that the Author conceived it a duty to give the world gratuitously those instructions which he had received from motives of friendship, and to strengthen them with very few facts, compared to the number he might have offered: requesting an an impartial consideration of his labour and a reciprocal readiness of communication in his Brethren.

Though the Medical world have perhaps relaxed a little in their terrors relative to evacuations in gouty people, yet the practice of giving bark in such large and repeated doses is, I believe, new in England. The Author's reflections also relative to the analogy between that disease and intermittent fevers, deserve notice, for though generally intermittents arise from a peculiar miasma, yet other causes will produce them. Other topical pains also have shown regular intermissions, and been cured by bark. But the extensive field of observation which our Author possessed, entitles him to particular attention in his remark, that gouty people, rarely, if ever, become the subjects of ague, and that in damp, aguish situations, gout is most prevalent. Professor Tavares' office as *Archiatro*, gives him the most extensive correspondence; and it is well known, that the country along the banks of the Tagus for a considerable extent in Portugal, is marshy and aguish. I might add, as a further analogy, that during my eight years residence in the delightful Island of Madeira, I saw no case of ague, nor scarcely of gout, among any of the natives; nor is there any swampy ground in any of the inhabited part of the Island. This hint is thrown out, that the readers of your extensively circulated Work, may improve it by observations on the air, situation, and diseases, of their respective circles of Practice.

I am, &c.

*London, June,* 1804. JOSEPH ADAMS.

